# MiRNA-128-3p Restrains Malignant Melanoma Cell Malignancy by Targeting NTRK3

**DOI:** 10.3389/fonc.2020.538894

**Published:** 2021-01-26

**Authors:** Xinxin Zhou, Jiayuan He, Qingyuan Wang, Teng Ma

**Affiliations:** ^1^ Academy of Traditional Chinese Medicine, Liaoning University of Traditional Chinese Medicine, Shenyang, China; ^2^ Department of Neurobiology, School of Life Sciences, China Medical University, Shenyang, China

**Keywords:** miR-128-3p, malignant melanoma, NTRK3, proliferation, migration, invasion, apoptosis, post-transcriptional regulation

## Abstract

The functions of non-coding RNA, including microRNA (miRNA), have attracted considerable attention in the field of oncology, In this report, we examined the roles and molecular mechanisms of miR-128-3p, as related to the biological behaviors of malignant melanoma (MM). We found that miR-128-3p was expressed in low levels in these MM cells and may serve as a tumor suppressor by inhibiting proliferation, migration, and invasion, as well as inducing apoptosis in these MM cells. Moreover, neurotrophin receptor 3 (NTRK3), which serves as an oncogene that can enhance malignant behaviors of MM cells, was up-regulated in MM cells. Our current survey disclosed a complementary binding between miR-128-3p and the NTRK3 3′ untranslated regions (3′-UTR), while luciferase activities of NTRK3 3′-UTR were restrained by miR-128-3p in 293T cells. The effects of pre-miR-128-3p and sh-NTRK3 as well as anti-miR-128-3p and NTRK3(+) appeared to function synergistically in producing malignant progression. Moreover, there were possible to have counteracted effects for pre-miR-128-3p and NTRK3(+) in malignant progression. These findings established that miR-128-3p can function as a tumor suppressor by inhibiting carcinogenesis of the oncogene, NTRK3. Collectively, miR-128-3p and NTRK3 genes participate in modulating the malignant behavior of MM, and may represent new therapeutic targets for MM.

## Introduction

Cutaneous malignant melanoma (MM), which originates from melanocytes in the epidermis, is a highly aggressive and the most lethal form of skin cancer worldwide (1). The incidence of MM has been increasing of late, showing one of the highest rates of cancer-related diseases (2). Treatment of MM mainly consists of surgical excision and chemotherapy, however MM is notoriously resistant to chemotherapy. Although treatment options have greatly improved in recent years, the high metastasis rate of MM, results in a 5-year survival rate of only 5–10% (3–5). Accordingly, treatments to improve the prognosis of MM represent an important issue.

Interestingly, both melanocytes and the nervous system originate from the neuroectoderm and express neurotrophins (NTs) (6). When NTs bind to neurotrophin receptors (NTRs), these receptors undergo dimerization and autophosphorylation, which facilitates survival and differentiation of neuron (7). These proto-oncogene NTRs are involved in the genesis of neurogenic or neuroendocrine tumors (8, 9). A NTR of significance to this report is that of neurotrophin receptor 3 (NTRK3), which belongs to the neurotrophin receptor Trk family. NTRK3 is transmembrane receptor tyrosine kinase and binds to neurotrophin-3 (NT-3) (10). Results from *in vitro* experiments have revealed that human epidermal melanocytes express NTRK3 and the p75 NTR, a neurotrophin receptor known to bind all NTs with equal, but low, affinity (11). Moreover, p75NTR and NTRK3 are highly expressed in MM cells (1, 12) and P75 NTR receptor signaling contributes to promoting proliferation and migration of MM cells as show in MM cell lines (1). However, to the best of our knowledge, the role of NTRK3 in human MM has not been elucidated. In this study, the regulatory mechanisms of NTRK3 on MM were assessed.

MicroRNAs (miRNAs) are a class of non-coding RNAs with 18–25 nucleotides. MiRNAs can inhibit the expression of target genes at the post transcriptional level by binding to 3′ untranslated regions (3′-UTR) of the target gene (13). As a result, they are critically related to many biological processes such as proliferation, differentiation, apoptosis, and metastasis (14). In particular, miRNAs may act as an oncogene and a tumor inhibitor gene due to their aberrant expression within normal tissues (15, 16). One specific miRNA of relevance to this study is that of, miR-128-3p, which is enriched in the brain and is known to act as a tumor-suppressor of glioma and bladder cancer (17–19). Conversely, miR-128-3p can also serve as an oncogene in hepatocellular carcinoma and T-cell acute lymphoblastic leukemia (20, 21). In some preliminary work within our laboratory we found that low-expressions of miR-128-3p were present in MM cells. Consequently, we speculated that there may be a relationship between miR-128-3p and NTRK3, and, in fact, there is a binding site of miR-128-3p at 209-215 bp of NTRK3 3’-UTR as identified using TargetScan 7.0 software. When collating this information, we hypothesized that miR-128-3p may play a key role in the tumorigenesis and development of MM. However, the expression of miR-128-3p and signaling pathways modulated by miR-128-3p as well as its relationship with NTRK3 that might be involved with influencing the malignant behaviors of MM are not very clear.

Therefore, in this report we examined the expression and function of miR-128-3p and NTRK3 in MM cells and found that miR-128-3p and NTRK3 proved to be tumor suppressor and oncogenes of MM cells, respectively. In addition, the mechanisms of miR-128-3p involved with regulating NTRK3 expression and the influence of miR-128-3p/NTRK3 on MM biological processes were studied. Overall, we found that miR-128-3p/NTRK3 may play critical roles in the progression of malignancy in MM and may serve as an important new treatment targets for this condition.

## Materials and Methods

### Cell Lines and Cultures

The human embryonic kidney (HEK 293T) and MM (A375, M14, MeWo, and SK-MEL-5) cell lines were obtained from American Type Culture Collection (ATCC) (Rockville, Maryland, MD, USA). They were cultured with Dulbecco’s Modified Eagle Medium (high glucose) containing 10% fetal bovine serum (Gibco, Carlsbad, CA, USA) in a constant temperature incubator (37°, 5% CO_2_). Normal human epidermal melanocytes (NHEMs) were obtained from Lonza (Walkersville, MD, USA) and cultured in Melanocyte Cell Basal Medium-4 supplemented with the MGM-4 Bullet Kit (Lonza, Walkersville, MD, USA) in a constant temperature incubator (37°, 5% CO_2_).

### Quantitative Real Time PCR (qRT-PCR)

The extraction and quantification of mRNA were performed according to procedures described in our previous study (13). The PrimeScriptTM RT reagent Kit (TaKaRa, Dalian, China) was used to reverse transcript mRNA into cDNA according to the instructions provided. Expressions of miR-128-3p and NTRK3 were assessed with use of SYBR^®^Premix Ex Taq II (TaKaRa, Dalian, Liaoning, China) according to instructions. U6 and GAPDH served as reference genes. All primers were synthetized by Lixin (Shenyang, Liaoning, China), with the sequence information contained in **Table 1**. The relative expressions of miR-128-3p and NTRK3 were calculated as relative quantitative values after normalizing with reference genes.

**Table 1:** Oligonucleotides used for qRT-PCR, their Amplicon Size, GenBank accession number, and annealing temperatures.

Gnen	Sequence	Amplicon size	Genbank accession number	Annealing temperature
NTRK3	(F) 5’TCCAGAGTGGGGAAGTGTCT-3’ (R) 5’-CCATGGTTAAGAGGCTTGA-3’	89	NM_001007156	58°C
GAPDH	(F) 5’-AAATCCCATCACCATCTTCCAG-3’ (R) 5’-TGATGACCCTTTTGGCTCCC-3’	149	NM_002046.4	58°C

### Cell Transfection of miRNAs

The agomir and antagomir for miR-128-3p (pre-miR-128-3p and anti-miR-128-3p) as well as their negative control (pre-miR-con and anti-miR-con) were obtained from GenePharma (Shanghai, China). Lipofectamine 3000 (Invitrogen, Foster City, CA, USA) was applied to transfect miRNAs into A375 and M14 cells according to manufacturers’ protocols. The transfected efficiency was evaluated with use of qRT-PCR, with 48 h after transfection being the best time point for subsequent examinations.

### Transfection and Generation of Stable Cell Lines

The expression plasmid [NTRK3(+)] (truncated isoform of NTRK3, Genbank accession number NM_001007156) and negative control plasmid (NTRK3(+)-NC) were designed and manufactured by GenScript (Nanjing, Jiangsu, China). The silenced plasmid for NTRK3 (sh-NTRK3) and its negative control plasmid (sh-NTRK3-NC) were designed and manufactured by GenePharma (Shanghai, China). Lipofectamine 3000 (Invitrogen, Foster City, CA, USA) was applied to transfect vectors into A375 and M14 cells according to instructions. G418 (Sigma-Aldrich, St Louis, MO, USA) was used to screen stable transfected cell lines according to procedures described in our previous study (13).

### Cell Proliferation Assay

A375 and M14 cells (2 × 10^3^ per well) were seeded into a 96-well plate. A CCK-8 dilution (Beyotime, Nantong, Jiangsu, China) consisting of 10 μl was then added to each well after 48 h. A SpectraMax M5 microplate instrument (Molecular Devices, Sunnyvale, CA, USA) was used to assess the absorbance at 450 nm, which represented cell proliferation ability.

### Cell Apoptosis Detection

The Annexin V-PE/7-AAD staining apoptosis kit (KeyGEN, Nanjing, Jiangsu, China) was used to evaluate apoptosis of A375 and M14 cells according to procedures described in our previous study (22). The apoptosis rate was assessed using CELLQuest software (BD Biosciences, San Jose, CA, USA).

### Cell Migration and Invasion Assay

MM cells (1 × 10^5^ per well) were added to the medium (serum-free) in the upper compartment of the 24-well transwell, while 500 μl medium (10% FBS) per well was added to the lower compartment. The migratory and invasive ability of MM cells were examined according procedures described in our previous study (23). As viewed under a microscope, cells invading the inferior side of the membrane in 5 randomly selected fields were counted. Generally speaking, we calculated the total number of cells in the upper left, lower left, upper right, lower right, and center of the field of view, and then divided by 5 to get the average value. The average value of transmembrane A375 and M14 cells obtained by the above method represented the migration and invasion ability of the cells.

### Cell Fractionation and Western Blot Analysis

Protein extraction and quantification were performed according to procedures described previously in our laboratory (13). The protein lysate (40 μg) was separated by polyacrylamide gel electrophoresis and then transferred onto a polyvinylidene fluoride membrane. The membranes were blocked with 5% skim milk powder for 2 h, hybridized overnight at 4°C with the NTRK3 antibody (Upstate, New York, USA) or GAPDH (Santa Cruz Biotechnology) and incubated at room temperature for 2 h with the secondary antibody. As the antibody is directed against the entire extracellular domain of the NTRK3 receptor, corresponding to amino acid residues 1-429, the epitope of the NTRK3 antibody is against both the full length (150 kDa) and truncated isoform (50 kDa). In preliminary experiments performed in our laboratory, no significant differences in the full-length form expression were obtained in A375 and M14 cells as compared with that of NHEMs (Data not shown). Only the expression of the truncated form (50 kDa) of the NTRK3 protein was determined with use of western blot. The FluorChem M imaging analysis system (Protein Simple, San Jose, CA USA) was used to scan the protein bands and calculate relative expressions of the NTRK3 protein.

### Dual-Luciferase Reporter Assay

The target genes of miR-128-3p were predicted with use of MiRDB and Targetscan (http://www.mirdb.org/miRDB/ and http://www.targetscan.org/). The NTRK3 3’-UTR wild-type luciferase reporter plasmid (NTRK3 wt), mutant plasmid (NTRK3 mut) and a negative control plasmid (NC, carrying non-targeting sequence) were constructed by GenePharma (Shanghai, China). The miRNAs (pre-miR-128-3p or pre-miR-con) and plasmids (NTRK3 wt and NTRK3 mut) were co-transfected into HEK 293T cells by means of Lipofectamine 3000. Luciferase activity was determined with use of the dual luciferase reporter gene assay system (Promega, Madison, WI, USA) at 48 h after transfection.

### Statistical Analysis

Each experiment was repeated three times and the data are presented as the means ± SDs. T*-*tests or one-way ANOVAs were used to determine whether statistically significant differences were present between/among the groups. Bonferroni post-test was used if the ANOVA was significant. A *P <*0.05 was required for results to be considered as statistically significant.

## Results

### MiR-128-3p Was Down-Regulated in MM Cells

**Figure 1:**
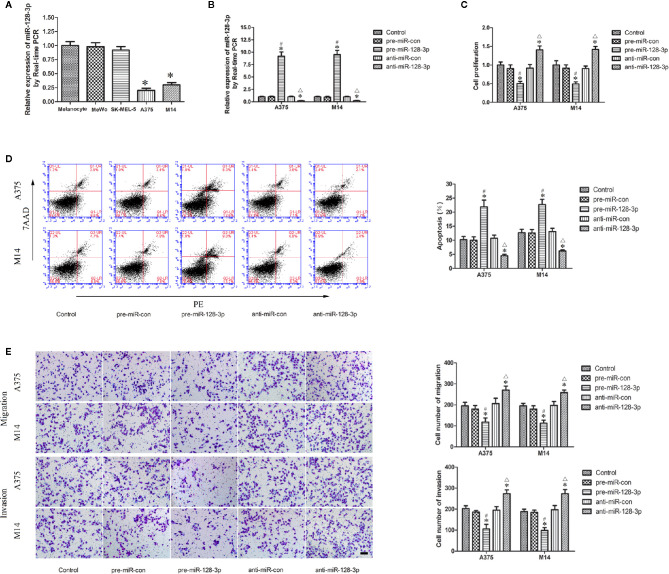
Expression and function of miR-128-3p in MM cells. **(A)** Relative expression of miR-128-3p as detected with qRT-PCR. U6 RNA level was used as an internal control. Values are means ± SDs (n = 10/group). *P < 0.05 vs. melanocyte group. **(B)** Transfection efficiency for pre-miR-128-3p and anti-miR-128-3p as detected with qRT-PCR. pre-miR-128-3p and anti-miR-128-3p were transfected into MM cells. U6 RNA level was used as an internal control. Values are means ± SDs (n = 5/group). *P < 0.05 vs. control group; ^#^ *P* < 0.05 vs. pre-miR-con group; ^△^ *P* < 0.05 vs. anti-miR-con group. **(C)** CCK8 assay to evaluate effects of miR-128-3p on MM cell proliferation. **(D)** Flow cytometry was used to analyze effects of miR-128-3p on apoptotic ratios of MM cells. **(E)** Transwell assays were used for assessing migration and invasion of MM cells with an altered expression of miR-128-3p. Representative images and accompanying statistical plots are presented. For panels **(C–E)** Values are means ± SDs (n = 3/group). *P < 0.05 vs. control group; ^#^ *P* < 0.05 vs. pre-miR-con group; ^△^ *P* < 0.05 vs. anti-miR-con group. Scale bars represent 20 μm.

Compared with that observed in NHEMs, miR-128-3p expression was significantly decreased in A375 and M14 cells as revealed with qRTPCR analysis (**Figure 1A**), but no significantly changes were observed in MeWo and SK-MEL-5 cells, thereby using A375 and M14 cells as research objects in subsequent experiments.

### MiR-128-3p Functions as a Tumor Suppressor in MM Cells

Having identified the expression of miR-128-3p in A375 and M14 cells, we next examined the effects of miR-128-3p on the biological behaviors of MM cells. The expression of miR-128-3p displayed a nearly 10-fold up-regulation in the pre-miR-128-3p versus pre-miR-con group (**Figure 1B**). Following anti-miR-128-3p transfection, the expression of miR-128-3p was significantly down-regulated by nearly 0.2-fold as compared with that in the control groups (**Figure 1B**). Results from the CCK-8 assay showed that the proliferation of A375 and M14 cells in the pre-miR-128-3p group was decreased as compared with that observed in the control and pre-miR-con groups (**Figure 1C**), whereas a marked increase in cell proliferation was obtained in the anti-miR-128-3p group as compared with that of the control and anti-miR-con groups (**Figure 1C**). Results from the flow cytometry analysis suggested that the over-expression of miR-128-3p markedly promoted apoptosis of A375 and M14 cells as compared with that of the control and pre-miR-con groups (**Figure 1D**), whereas cell apoptosis in the anti-miR-128-3p group was significantly decreased as compared with that observed in the control and anti-miR-con groups (**Figure 1D**). Results from the transwell assays implied that the number of migrated and invaded cells were significantly decreased in the pre-miR-128-3p versus control and pre-miR-con groups (**Figure 1E**), while the number of migrated and invaded MM cells was increased in the anti-miR-128-3p group as compared with that of the control and anti-miR-con groups (**Figure 1E**). Proliferation, apoptosis rates, and the ability for migration and invasion were not significantly different among the control, pre-miR-con and anti-miR-con groups (**Figures 1C–E**). These findings demonstrate that miR-128-3p can serve as a tumor suppressor in the malignant progression of MM.

### NTRK3 Was Up-Regulated in MM Cells

**Figure 2:**
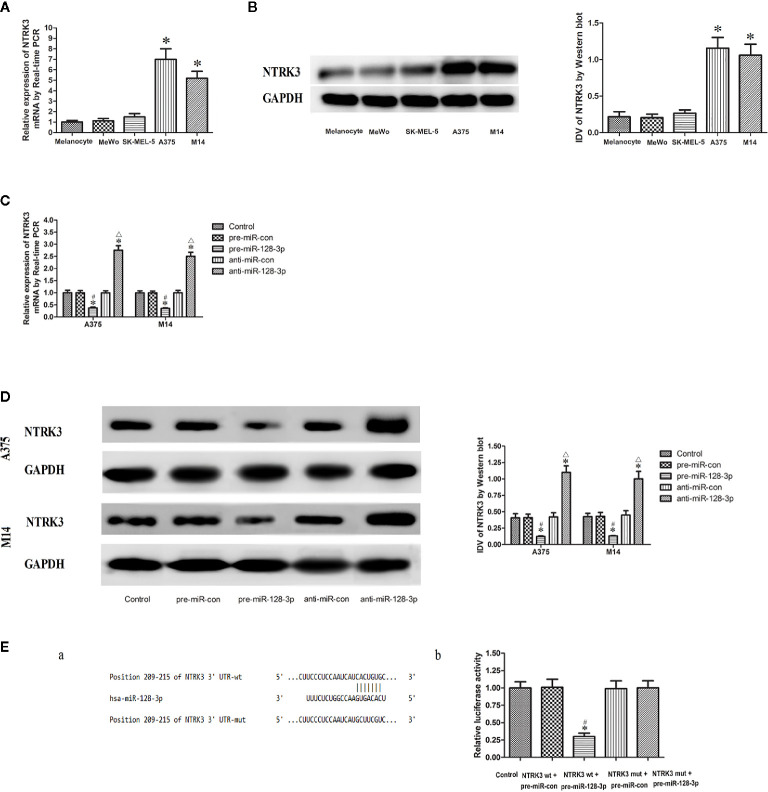
NTRK3 is a direct target gene of miR-128-3p. **(A)** Relative mRNA expressions of NTRK3 as determined with qRT-PCR. GAPDH RNA levels were used as an endogenous control. Values are means ± SDs (n = 10/group). *P < 0.05 vs. melanocyte group. **(B)** Protein expressions of NTRK3 as assessed with Western blot. Values are means ± SDs (n = 3/group). *P < 0.05 vs. melanocyte group. **(C)** qRT-PCR analysis of the effects of miR-128-3p on NTRK3 mRNA expression in MM cells. **(D)** Western blot was performed to analyze the effects of miR-128-3p on NTRK3 protein expression in MM cells. Representative images and accompanying statistical plots are presented. For panels C and D, Values are means ± SDs (n = 3/group). *P < 0.05 vs. control group; ^#^ *P* < 0.05 vs. pre-miR-con group; ^△^ *P* < 0.05 vs. anti-miR-con group. **(E)** Luciferase activity of NTRK3 3′-UTR as detected after co-transfection. Relative luciferase activity was expressed as firefly/renilla luciferase activity. Putative binding sites of NTRK3 3′-UTR as matching with the seed region of miR-128-3p was predicted with TargetScan. Values are means ± SDs (n = 3/group). *P < 0.05 vs. control group; ^#^ *P* < 0.05 vs. NTRK3wt.pre-miR-con group.

As based on an online bioinformatic analysis, it was predicted that NTRK3 might be a target gene of miR-128-3p. Therefore, qRTPCR and western blotting were used, with the results that the expression of NTRK3 was significantly elevated in A375 and M14 cells as compared with that of NHEMs (**Figures 2A, B**). Conversely, no significantly changes in NTRK3 expression were obtained in MeWo and SK-MEL-5 cells. Therefore, Subsequent experiments were only performed in A375 and M14 cells.

### MiR-128-3p Negatively Regulates NTRK3 Expression

NTRK3 mRNA and protein expressions were down-regulated in the pre-miR-128-3p as compared to that of the control and pre-miR-con groups (**Figures 2C, D**). In contrast, transfection with anti-miR-128-3p increased the expression of NTRK3 mRNA and protein as compared with that observed in its control groups (**Figures 2C, D**). No statistically significant differences were obtained among the control, pre-miR-con and anti-miR-con groups (**Figures 2C, D**). These finding provided further evidence that miR-128-3p is regulating the expression of NTRK3.

### NTRK3 Is a Direct Target Gene of MiR-128-3p

With use of TargetScan software 7.0 it was forecasted that there is a binding site of miR-128-3p at 209-215 bp of NTRK3 3’-UTR (**Figure 2E.a**). The seed for miR-128-3p to NTRK3 3’-UTR is shown in **Figure 2 E.a**. NTRK3 was a direct target gene of miR-128-3p as revealed from results of the luciferase assay. In accord with these expectations, a co-transfection with NTRK3 wt and pre-miR-128-3p, but not NTRK3 wt and pre-miR-con, significantly reduced the relative luciferase activity as compared with that of control (**Figure 2E.b**). To further verify whether miR-128-3p directly targeted NTRK3 through the predicted binding site, a luciferase reporter vector containing the Mut-type 3′-UTR of NTRK3 (NTRK3-3′UTR-Mut) was constructed. Co-transfection of NTRK3 mut and pre-miR-128-3p did not affect the relative luciferase activity. Taken together, these results demonstrate that miR-128-3p can bind NTRK3 3’-UTR and NTRK3 was a target gene of miR-128-3p.

### NTRK3 Is an Oncogene in MM Cells

**Figure 3:**
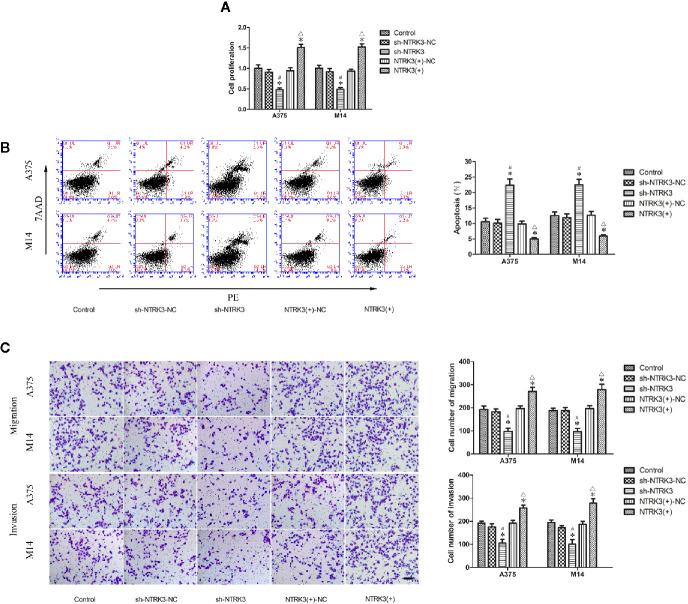
NTRK3 is an oncogene in MM cells. **(A)** CCK8 assay was used to evaluate effects of NTRK3 on the proliferation of MM cells. **(B)** Flow cytometry was used to detect apoptosis of MM cells after overexpression or silencing of NTRK3. **(C)** Transwell assays were performed to assess migration and invasion of MM cells under conditions of altered NTRK3 expression. Representative images and accompanying statistical plots are presented. For panels **(A–C)** Values are means ± SDs (n = 3/group). *P < 0.05 vs. control group; ^#^ *P* < 0.05 vs. sh-NTRK3-NC group; ^△^ *P* < 0.05 vs. NTRK3(+)-NC group. Scale bars represent 20 μm.

After verifying that NTRK3 was up-regulated in MM cells and was direct target of miR-128-3p, we next attempted to elucidate some of the effects of NTRK3 on the biological behaviors of MM cells. CCK-8 assay results indicated that the proliferation of A375 and M14 cells in the sh-NTRK3 group was significantly decreased compared with that of the control and sh-NTRK3-NC groups (**Figure 3A**), while proliferation in the NTRK3(+) group was significantly increased compared with the control and NTRK3(+)-NC groups (**Figure 3A**). A down-regulated expression of NTRK3 (sh-NTRK3) resulted in an increased apoptotic ratio of A375 and M14 cells as compared with that in the control and sh-NTRK3-NC groups (**Figure 3B**), whereas an up-regulation of NTRK3 [NTRK3(+)] significantly suppressed apoptosis in A375 and M14 cells as compared with the control and NTRK3(+)-NC groups (**Figure 3B**). Transwell assays were conducted to assess the effects of NTRK3 on the migratory and invasive ability of A375 and M14 cells. As predicted, the migratory and invasive ability in the sh-NTRK3 group was reduced compared with that of the control and sh-NTRK3-NC groups (**Figure 3C**), whereas increased migratory and invasive capacities were observed in the NTRK3(+) versus the control and NTRK3(+)-NC groups (**Figure 3C**). No statistically significant differences were obtained among the control, sh-NTRK3-NC and NTRK3(+)-NC groups (**Figures 3A–C**). Collectively, these results showed that NTRK3 can be considered as an oncogene in A375 and M14 cells.

### MiR-128-3p Exerts Tumor Suppression by Inhibiting Carcinogenesis of the Oncogenic NTRK3

**Figure 4:**
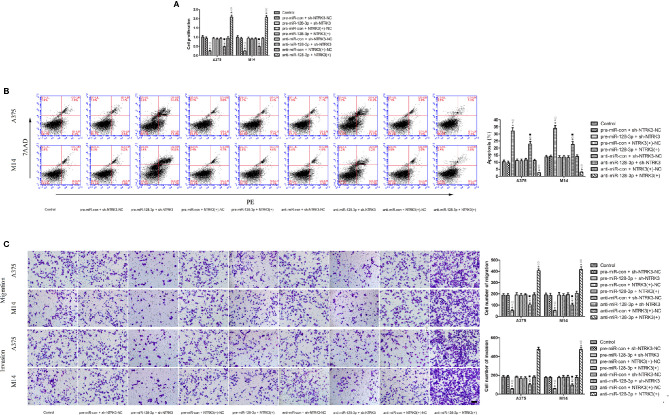
MiR-128-3p exerted cell proliferation suppression by inhibiting carcinogenesis of the oncogenic NTRK3 gene. **(A)** CCK-8 assay was used to assess effects of miR-128-3p and NTRK3 on proliferation of MM cells. Values are means ± SDs (n = 3/group). *P < 0.05 vs. control group; ^#^ *P* < 0.05 vs. pre-miR-con + sh-NTRK3-NC group. ^□^ *P* < 0.05 vs. the other groups. **(B)** Flow cytometry was used to determine effects of miR-128-3p and NTRK3 on apoptosis of MM cells. Values are means ± SDs (n = 3/group). *P < 0.05 vs. control group; ^△^ *P* < 0.05 vs. anti-miR-con + sh-NTRK3-NC group; ^■^ *P* < 0.05 vs. the other groups. **(C)** Transwell assays were employed to detect effects of miR-128-3p and NTRK3 on migration and invasion of MM cells. Representative images and accompanying statistical plots are presented. Values are means ± SDs (n = 3/group). *P < 0.05 vs. control group; ^▽^ *P* < 0.05 vs. anti-miR-con + NTRK3(+)-NC group; ^♢^ *P* < 0.05 vs. the other groups. Scale bars represent 20 μm.

Having established that miR-128-3p and NTRK3 functioned as a tumor suppressor and an oncogene, respectively, and that NTRK3 was a target of miR-128-3p, the next issue to address was whether miR-128-3p exerted a tumor inhibitor effect by hindering carcinogenesis of the oncogenic NTRK3. To accomplish this goal, pre-miR-128-3p or anti-miR-128-3p were transfected into stable NTRK3(+) or sh-NTRK3 MM cells prior to assessing their malignant biological behavior. Results from the CCK8 assay indicated that proliferation of A375 and M14 cells was remarkably reduced in the pre-miR-128-3p + sh-NTRK3 group as compared with all other groups (**Figure 4A**). Similarly, with the exception of the pre-miR-128-3p + sh-NTRK3 group, A375 and M14 cell proliferation in the anti-miR-128-3p + sh-NTRK3 group was dramatically reduced compared to all other groups, while the proliferation of A375 and M14 cells in the anti-miR-128-3p + NTRK3(+) group was remarkably elevated in comparison with all other groups (**Figure 4A**). No statistically significant differences were observed among the control, the pre-miR-con + sh-NTRK3-NC, pre-miR-con + NTRK3(+)-NC, pre-miR-128-3p + NTRK3(+), anti-miR-con + sh-NTRK3-NC, and anti-miR-con + NTRK3(+)-NC groups (**Figure 4A**). Moreover, A375 and M14 cells treated with pre-miR-128-3p + sh-NTRK3 exhibited increased apoptotic ratios as compared with that obtained in all other groups (**Figure 4B**). With the exception of the pre-miR-128-3p + sh-NTRK3 group, higher rates of apoptosis were observed in A375 and M14 cells within the anti-miR-128-3p + sh-NTRK3 group as compared with all other groups, whereas apoptosis was dramatically suppressed in NTRK3(+) cells with anti-miR-128-3p transfection versus that observed in all other groups (**Figure 4B**). No statistically significant differences were detected among the control, the pre-miR-con + sh-NTRK3-NC, pre-miR-con + NTRK3(+)-NC, pre-miR-128-3p + NTRK3(+), anti-miR-con + sh-NTRK3-NC, and anti-miR-con + NTRK3(+)-NC groups (**Figure 4B**). In addition, the migration and invasion of MM cells demonstrate the same tendency as their proliferation, and the number of A375 and M14 cells showing migration and invasion were remarkably reduced in the pre-miR-128-3p + sh-NTRK3 group as compared with all other groups (**Figure 4C**). As predicted, with the exception of the pre-miR-128-3p + sh-NTRK3 group, the migratory and invasive ability within the anti-miR-128-3p + sh-NTRK3 group was attenuated versus that of all the other groups, while the number of A375 and M14 cells showing migration and invasion were dramatically increased in the anti-miR-128-3p + NTRK3(+) group as compared with all other groups (**Figure 4C**). There were no statistically significant differences among the control, pre-miR-con + sh-NTRK3-NC, pre-miR-con + NTRK3(+)-NC, pre-miR-128-3p + NTRK3(+), anti-miR-con + sh-NTRK3-NC, and anti-miR-con + NTRK3(+)-NC groups (**Figure 4C**). These findings indicate that miR-128-3p exerted a tumor suppression role by inhibiting carcinogenesis of the oncogenic NTRK3.

## Discussion

Our present results reveal that a low expression of miR-128-3p is observed in MM cells and this reduction in miR-128-3p expression promoted cell proliferation, migration, and invasion, but inhibited apoptosis in MM cells. Moreover, miR-128-3p was found to function as a tumor suppressor in MM cells. Unlike miR-128-3p, NTRK3 was up-regulated in MM cells and acted as an oncogene, enhancing malignant behaviors of MM cells. Our current results delineated the complementary binding between miR-128-3p and 3′-UTR of NTRK3, which could prohibit NTRK3 expression in MM cells. Finally, our results indicated that miR-128-3p can function as a tumor suppressor by inhibiting carcinogenesis of the oncogenic NTRK3. In this way, we provide the first evidence that miRNA-128-3p restrains MM cell malignancy by targeting NTRK3.

MM is one of the most lethal tumors whose incidence has been increasing worldwide (5, 24). In spite of great progresses in the early diagnosis and treatment of MM, the prognosis continues to be poor (25–27). Accordingly, the identification of novel therapies for MM are sorely needed. Increasing evidence has been presented showing that miRNAs are associated with the malignant progression of MM (2, 3). For example, Guo et al. found that miR-23a knockdown promoted cutaneous melanoma metastasis by activating autophagy (24), while Wang et al. reported that an over-expression of miRNA-136 produced a down-regulation of the Wnt signaling pathway by targeted binding to PMEL. Such an effect would restrain EMT, proliferation, migration, and invasion, as well as promote apoptosis in melanoma cells (28). And, Liu et al. documented that miR-499a-5p inhibited LMX1A-mediated malignant behavior by directly targeting LMX1A-3′-UTR in glioma cells (29). As we all know, malignant melanoma is a heterogeneous disease and constitutes a varied mix of many cell types (30, 31). So, we studied the expression and function of miRNA-128-3p/NTRK3 in A375, M14, MeWo, and SK-MEL-5 cell lines. Our results affirmed that significantly changes in expression of miR-128-3p/NTRK3 were observed in A375 and M14 cells. Conversely, no significantly changes were obtained in MeWo and SK-MEL-5 cells. These results presented that miR-128-3p/NTRK3 had nothing to do with the malignant biology of MM in MeWo and SK-MEL-5 cells. Therefore, Subsequent experiments were only performed in A375 and M14 cells, and A375 and M14 cells were used as the research object.

MiRNAs are considered as a class of gene regulators that can modulate many biological processes (32). Accumulating evidence has indicated that aberrant expression of endogenous miRNAs leads to regulatory abnormalities and dysfunctions that can contribute to the pathogenesis of many types of tumors (19, 33). The miRNA under investigation in this report, miR-128-3p, is a brain-rich miRNA encoded by two individual genes, miR-128a and miR-128b, which encode the same mature sequence (34). Results from previous studies have shown that miR-128-3p was down-regulated in several cancers including bladder cancer, glioma, esophageal squamous-cell cancer, colorectal carcinoma, and hepatocellular carcinoma (17–19, 35, 36). Consistent with these findings, our current data also demonstrated a decrease in the expression of miR-128-3p in MM. However, it should be noted that there exist reports indicating that miR-128-3p expression is increased in gastric and breast cancers (33, 37), which is contrary to our current results. Such seemingly inconsistent findings may be the result of different roles exerted by miR-128-3p within specific types of tumors. Overall, when combining the results of these previous reports with our current findings, it appears that changes in miR-128-3p expression might be essential for the occurrence and malignant progression of MM.

The role of miR-128-3p in malignant progression of MM was further investigated. As predicted, our data showed that an up-regulation of miR-128-3p restrained the proliferation, migration, and invasion of MM cells, as well as increased the rate of apoptosis of MM cells, while silencing of miR-128-3p showed the opposite effects. We also found that M14 and A375 cells demonstrated similar migratory and invasive rates as revealed from results of the transwell assay. Consistent with these findings were the results of Wen et al. reporting similar migratory abilities of M14 and A375 during the process of Rho GTPases influence on cytoskeletal and migratory activity (38). In this way, miR-128-3p serves as a tumor inhibitor in MM. Similarly, anti-tumor effects of miR-128-3p have been observed in several other types of tumors (17–19, 34, 36, 39–46). For example, Liu et al. reported that an over-expression of miR-128-3p, miR-101, and miR-27b inhibited VEGF-C secretion of gastric cancer cells, thereby blocking HUVEC migration, proliferation, and tubulation (44). Conversely, there are reports demonstrating that miR-128-3p facilitated the occurrence and development of some cancers. Notably, MiR-128-3p has been shown to be a new, robust oncogene candidate in T cell acute lymphoblastic leukemia (20), a result which differs from our current findings. One possible explanation may be due to the potential for tumors with different origins to exert diverse mechanisms involved with tumorgenesis. Taken together, it seems that miR-128-3p might function as a tumor suppressor in MM, which suggests a novel potential treatment protocol against MM.

Recently, increasing evidence has accrued showing that miRNAs, as modulaters of cancer, could affect the expression of critical genes connected with tumourigenesis and malignant behaviors (33, 45). MiRNAs restrain the expression of target genes with the aid of guiding a RNA induced silencing complex (RISC) to bind the 3’-UTR of mRNAs and seed sequence of miRNAs. This combination of events leads to an inhibition of translation or mRNA degradation (20). Our present data also reveal that the expression of NTRK3 was elevated, an effect which was modulated by miR-128-3p in MM cells. Others have reported that NTRK3 was up-regulated in MM, as well as in other types of tumors, as demonstrated both *in vivo* and *in vitro* (1, 12, 46–49). Here, we provide clear evidence that miR-128-3p modulates NTRK3 expression and was directly bound to NTRK3 3′-UTR at positions 209–215. As far as we know, miR-768-5p, miR-128-3p, miR-497, miR-765, miR-625, miR-509, and miR-485-3p all reduce luciferase activity of NTRK3 by targeting its 3′-UTR (29, 50–52). In addition, the findings from these reports indicate that miR-128-3p combines with NTRK3 3′-UTR through its seed region, which is consistent with our results (29, 50, 51). Therefore, these studies provide corroborative evidence that miR-128-3p could repress NTRK3 expression by directly combining with 3′-UTR of NTRK3.

NTRK3 is a classic tyrosine kinase receptor and consequently generally viewed as a protooncogene (53, 54). Our findings authenticated that the down-regulation of NTRK3 hinders proliferation, migration, and invasion ability, as well as producing an increase in the apoptosis of MM cells. These results were opposite to that observed in the miR-128-3p over-expression group. Our findings that NTRK3 was an oncogene in MM cells are in line with that of Kim et al. who reported that NTRK3 significantly enhanced the capability of breast cancer cells to bring about primary tumor formation and pulmonary metastases (49). However, contrary to our results, Xiong et al. reported that NTRK3 knockdown could promote migration, invasion and proliferation in SMMC7721 cells (32) and that NTRK3 induced apoptosis, and was believed to act as a tumor inhibitor gene in colorectal cancer (55, 56). These disparate functions of NTRK3 might be due to differences in pathological changes within different tumor tissues.

Mechanistically, miR-128-3p and NTRK3 have been identified as a tumor suppressor and an oncogene, respectively. Our current results provide some insights into the eventuality that miR-128-3p and NTRK3 can potentially exert reciprocal effects in the malignant progression of MM. MM cell proliferation and migration, as well as invasion, were dramatically attenuated in the pre-miR-128-3p + sh-NTRK3 group, while MM cells treated with pre-miR-128-3p + sh-NTRK3 exhibited increased apoptotic ratios as compared with that observed in the other groups. Moreover, MM cell proliferation and migration, as well as invasion, were dramatically enhanced in the anti-miR-128-3p + NTRK3(+) group, whereas NTRK3(+) cells co-transfected with anti-miR-128-3p dramatically suppressed apoptosis as compared with the other groups. These results suggest that synergistic effects may be present among pre-miR-128-3p and sh-NTRK3 as well as anti-miR-128-3p and NTRK3(+) with regard to malignant progression. No significant differences for the above test indicators were obtained in the pre-miR-128-3p + NTRK3(+) group, suggesting that these may have possibly counteracted the effects of pre-miR-128-3p and NTRK3(+) upon malignant progression. Taken together, the data provide robust evidence indicating that miR-128-3p can function as a tumor suppressor by inhibiting carcinogenesis of the oncogenic NTRK3.

In conclusion, our data suggest that miR-128-3p in MM cells could serve as a tumor inhibitor by negatively modulating malignant biological behaviors of MM cells. The findings that NTRK3 promoted malignant biological behaviors of MM cells, indicates that NTRK3 serves as an oncogene in MM cells. Combining our findings obtained with miR-128-3p and NTRK3, it appears that miR-128-3p exerts its tumor suppression by inhibiting the carcinogenesis of the oncogene, NTRK3. These results provided a novel and more comprehensive understanding of miR-128-3p and NTRK3 functions as related to MM. Accordingly, they can serve as a foundation for the development of new genetic diagnostic markers and targeted therapies involving miR-128-3p and NTRK3 for MM treatment.

## Data Availability Statement

All datasets generated for this study are included in the article.

## Author Contributions

XZ and TM conceived and designed the experiments. XZ, JH, and QW performed the experiments. QW and JH analyzed the data. XZ and TM drafted the manuscript. All authors contributed to the article and approved the submitted version.

## Funding

This work was supported by grants from the Natural Science Foundation of China (NO. 81803849).

## Conflict of Interest

The authors declare that the research was conducted in the absence of any commercial or financial relationships that could be construed as a potential conflict of interest.
